# Osteogenesis Imperfecta (Type IV) with Dental Findings in Siblings

**DOI:** 10.1155/2011/970904

**Published:** 2011-09-06

**Authors:** Shishir Ram Shetty, Deepa Dsouza, Subhas Babu, Preethi Balan

**Affiliations:** Department of Oral Medicine and Radiology, AB Shetty Memorial Institute of Dental Sciences, Nitte University, Mangalore 575018, India

## Abstract

Osteogenesis imperfecta (OI) is a hereditary disorder characterized by increased tendency for bone fractures due to high fragility. The clinical and radiological features of OI manifest in different age groups, although the disease is congenital in nature. Besides bone fragility, features like laxity of the ligaments, blue sclera, growth retardation, and scoliosis are also observed. In severe cases, respiratory distress and death have been reported. The most important oral finding in OI is the presence of yellowish-brown-coloured brittle teeth characteristic of dentinogenesis imperfecta. Genetic factors play a very important role in the pathogenesis of OI either as a dominant or recessive factor. When a child has OI, there is a 25% chance of the sibling to have the same disorder. We report two cases of OI in siblings born to parents with a history of consanguineous marriage. The clinical and radiological features of the two cases are described in detail.

## 1. Introduction

Osteogenesis imperfecta (OI) is a disorder characterized by bone fragility and with severe deformities [1]. OI exhibits a variety of clinical presentation ranging from intrauterine death to normal growth and low fracture incidence depending upon the severity [2]. OI also presents with features like skin and joint laxity but may not be pathognomic [2]. The basic pathophysiology for OI is the mutation in the gene coding for type I collagen [3].

The most widely accepted classification was proposed by Sillence et al. in 1979 [4]. The classification that had five broad categories based on clinical, radiological, and genetic factors [4]. Sillence's type IV is further subdivided into groups A and B, group B is characterized by the presence of dentinogenesis imperfect [4].

We report two cases of type IV B osteogenesis imperfecta in siblings with dental findings. 

## 2. Case Report

### 2.1. Case  1

**Figure 1:**
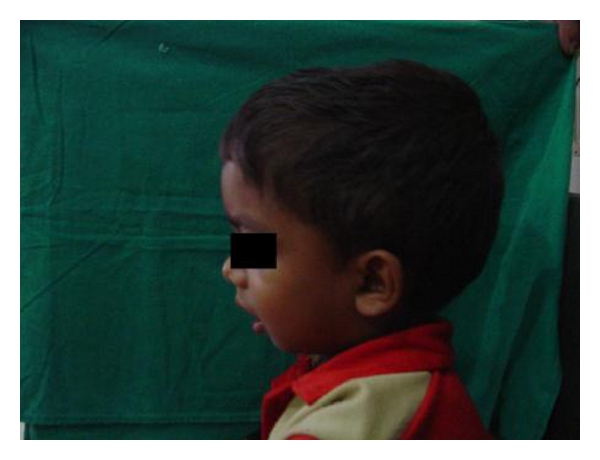
Clinical photograph of the patient showing brachycephalic head and frontal bossing.

**Figure 2:**
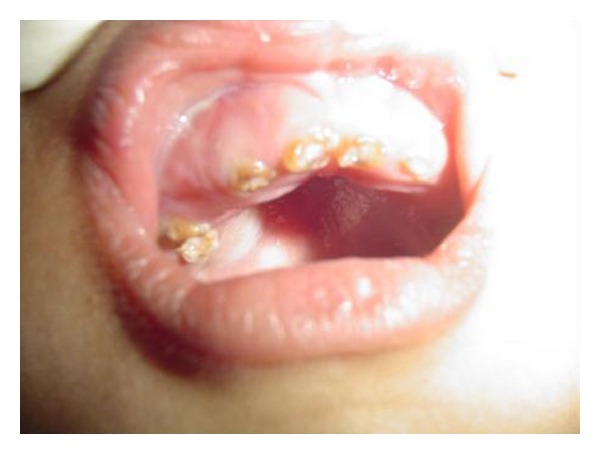
Intraoral photograph showing yellowish discolouration and chipping of the dentition.

**Figure 3:**
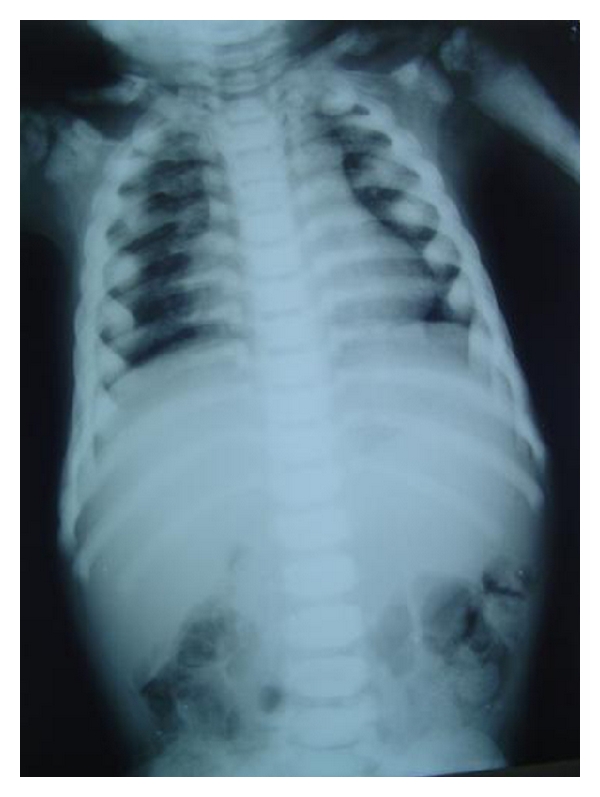
Chest radiograph showing long and narrow thorax (barrel-shaped chest) with anterior compression.

**Figure 4:**
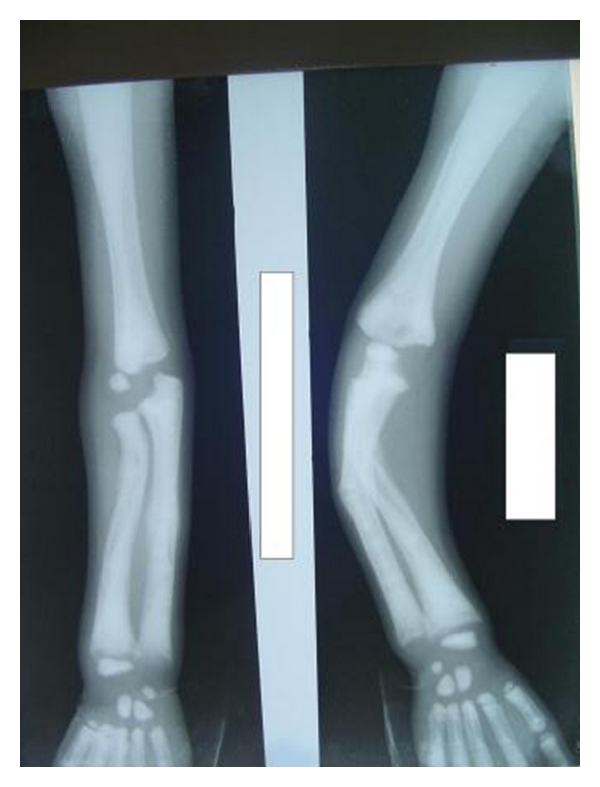
Radiograph of the upper limb showing bowing of the radius and ulnar.

A 4-year-old male child was brought to the general hospital with complaints of bowed upper and lower limbs. The patient had history of fractures due to minor trauma during the past 2-3 years. The patients elder sibling had similar problem. There was a history of consanguineous marriage. The fractures were treated by local specialists, but after treatment, bowing of the upper and lower limbs were noticed. The patient had growth deformity and abnormally large head with frontal bossing (Figure 1). After primary consultation with pediatricians and orthopaedicians a dental opinion for the discoloured teeth was advised. On intraoral examination, the teeth were yellowish to brownish in colour with complete chipping of the enamel was noted and delayed eruption of several deciduous teeth was also observed (Figure 2). Chest radiograph revealed long and narrow thorax with anterior compression (Figure 3). Radiograph of the upper limbs revealed malunion and bowing of radius and ulnar (Figure 4). Based upon these clinical findings and opinions from medical specialists, a provisional diagnosis of osteogenesis imperfect (Type IV B) with dentinogenesis imperfecta. Rickets and idiopathic juvenile osteoporosis were considered as differential diagnosis. The patient's elder brother was also called for examination.

### 2.2. Case  2

**Figure 5:**
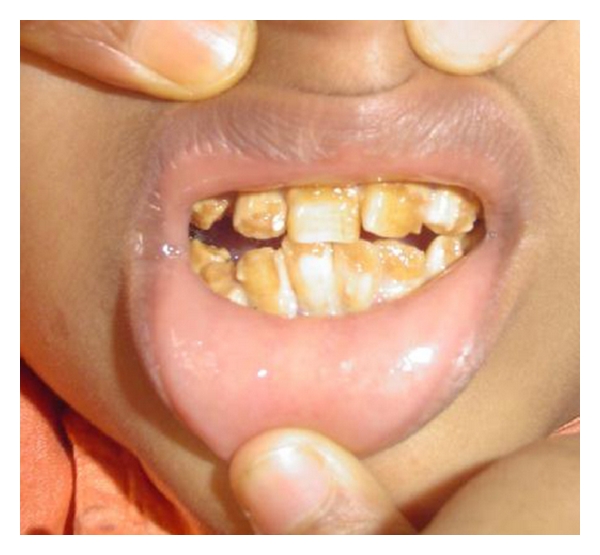
Intraoral photograph of the elder sibling showing yellowish discolouration of the dentition.

**Figure 6:**
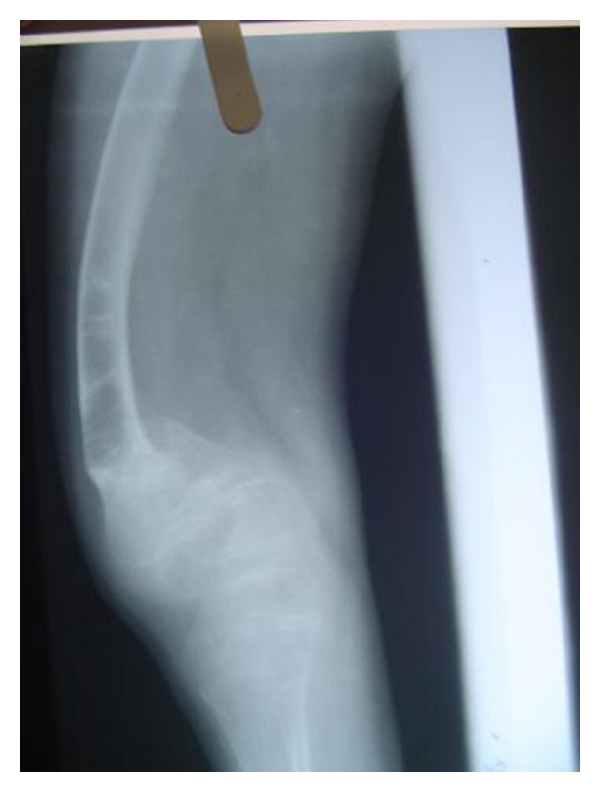
Radiograph of the lower limbs showing bowing of the femur, with widening of the metaphases.

**Figure 7:**
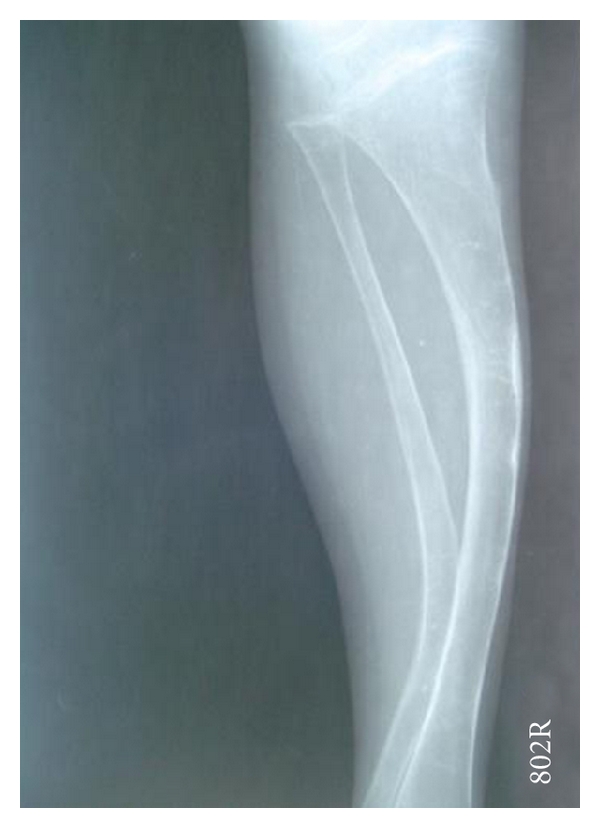
Radiograph showing bowing tibia and fibula.

Patient's elder brother who was 10 years old had a deformed growth with bowing in the upper and the lower limbs. He also reported of multiple fractures and frequent joint dislocations secondary to minor trauma. The dental findings were identical to that of the younger sibling, but there was no evidence of delayed eruption (Figure 5). Radiographs of the lower limbs revealed thinner cortices, bowing of the femur and tibia, and expanded metaphyses (Figures 6 and 7). Based on these features and the familial history, a diagnosis of osteogenesis imperfect (Type IV B) with dentinogenesis imperfecta was made. Because of logistical reasons, the patients could not continue the dental treatment in our institution.

## 3. Discussion

Osteogenesis imperfecta (OI), also known as “brittle bone disease”, is a heterogeneous group of genetic connective tissue-associated disorders [5]. Description of OI in medical literature dates back to 1678 [6]. The varying range of incidence has been reported from 1 : 10000 to 1 : 20000 births [7]. The most accepted classification of OI was proposed by Sillence et al. (1979) based on the phenotype and genotype [8, 9]. Mutations in the genes (COL1A1 and COL1A2) that encode the pro-alpha 1 and pro-alpha 2 polypeptide chains of type I collagen are responsible for most forms of OI. Thus, tissues like bone, dentin, sclera, and ligaments which are abundant Type I collagen are usually affected [5]. A reduction in the quantity of a structurally normal collagen results in OI Type I, whereas qualitative and quantitative alterations in the collagen synthesis result in OI Types II, III, and IV [10].

Three scenarios that occur to cause a child to be born with OI [11].

1. Direct inheritance from a parent: 50% chance of passing on the disorder to next generation.
2. A new dominant mutation: the gene spontaneously mutated in either the sperm or the egg before the child's conception.
3. Mosaicism: clinically unaffected parents with more than one affected child. The mutation occurred during the parent's fetal development.

Siblings in our case exhibited clinical feature of Sillence Type IV OI characterized by brittle bones, growth retardation, pathologic fractures, and DI. More severe forms of OI tend to manifest at a very young age and have poor survival rates. The moderate forms such as Sillence's Type IV has better survival rate as seen in our cases [2]. Sillence's Type IV OI is the most diversified of all the groups in the classification and cases which do not fit into the criteria from Type I to Type III are categorized under Sillence's Type IV. Type IV can be further classified into A and B [11]. Type IV A and B can be differentiated based on the dental findings, the former is associated with normal teeth and the later is associated with DI Type II. (DI) is associated with OI which is characterized by the mutation in the sialophosphoprotein gene [10]. Teeth affected present with an opalescent grayish brown hue. The enamel may be of normal thickness, but frequently is dislodged exposing the softer dentin which may be attributed to the smooth dentinoenamel junction [11]. Patients with osteogenesis imperfecta should be evaluated as soon as the deciduous teeth erupt with adequate dental treatment and oral hygiene instructions in order to reduce the need for extensive treatment [5].
